# The ‘Finnish new variant of *Chlamydia trachomatis*’ escaping detection in the Aptima Combo 2 assay is widespread across Norway, June to August 2019

**DOI:** 10.2807/1560-7917.ES.2019.24.42.1900592

**Published:** 2019-10-17

**Authors:** Tone Bjordal Johansen, Hilde Kløvstad, Rikard Rykkvin, Einar Bredo Herrfurth-Erichsen, Joakim Sorthe, Gro Njølstad, Marit Helen Ebbesen, Randi Monsen Nygaard, Ellen Kristin Sandmoen, Carina Thilesen, Annette Onken, Inger Liljedal, Ronza Hadad, Magnus Unemo

**Affiliations:** 1 Norwegian Institute of Public Health, Oslo, Norway; 2 European Public Health Microbiology Training Programme (EUPHEM), European Centre for Disease Prevention and Control (ECDC), Stockholm, Sweden; 3 Fürst Medical Laboratory, Oslo, Norway; 4 Haukeland University Hospital, Bergen, Norway; 5 Tønsberg Hospital Trust, Department of Medical Microbiology, Tønsberg, Norway; 6 Unilabs Laboratory Medicine, Department of Medical Microbiology, Skien, Norway; 7 Vestre Viken Hospital Trust, Department of Microbiology, Drammen, Norway; 8 Levanger Hospital, Department for Laboratory Medicine, Levanger, Norway; 9 WHO Collaborating Centre for Gonorrhoea and other STIs, National Reference Laboratory for STIs, Department of Laboratory Medicine, Faculty of Medicine and Health, Örebro University, Örebro, Sweden

**Keywords:** *Chlamydia trachomatis*, FI-nvCT, Aptima Combo 2 assay, diagnostic escape, Norway

## Abstract

The ‘Finnish new variant of *Chlamydia trachomatis*’ (FI-nvCT), escaping detection in the Aptima Combo 2 assay (AC2), is widespread across Norway. From June to August 2019, 84% (81/97) of available AC2/Aptima CT discordant samples from five laboratories were confirmed as FI-nvCT. Two additional CT variants (CT 23S rRNA C1514T and G1523A) also escaped AC2 detection. The high FI-nvCT proportion might indicate a long-term national spread and it cannot be excluded that FI-nvCT emerged in Norway.

A new variant of *Chlamydia trachomatis* (nvCT) was recently detected in Finland [1,2] and subsequently in Sweden [3]. This ‘Finnish nvCT’ (FI-nvCT), with a C1515T mutation in the 23S rRNA gene, causes false-negative or equivocal *C. trachomatis* (CT) test results in the nucleic acid amplification test (NAAT) Aptima Combo 2 (AC2) (Hologic Inc., San Diego, California, United States) [1-6].

Based on information from Finland [1,2], Sweden [3] and a rapid risk assessment published by the European Centre for Disease Prevention and Control (ECDC) on 17 June 2019 [4], we investigated the presence of possible and confirmed FI-nvCT cases [4] across Norway, in order to evaluate the need for recall of patients who may have received a false negative/equivocal AC2 CT test result.

## Confirmed cases of ‘Finnish new variant of *Chlamydia trachomatis’* are widespread across Norway

Aptima Combo 2 detects both CT (target: 23S rRNA) and *Neisseria gonorrhoeae* (target: 16S rRNA). The AC2 test results are measured in relative light units (RLUs) when run on the Panther or Tigris platform (Hologic), and the 23S rRNA C1515T mutation, located in the AC2 CT probe detection sequence, causes low RLU values, which are interpreted as negative or equivocal CT results [2,3,5]. The AC2 CT RLUs (when only a CT signal is detected) for negative results range from 1 to 24, for equivocal results from 25 to 99 and for positive results ≥ 100, as described by the manufacturer [1,6].

In Norway, six of the 17 laboratories diagnosing CT samples routinely use AC2, accounting for approximately 50% of the CT test volume across the country. Promptly after the finding of the FI-nvCT in Finland [1] and Sweden [3], and as advised by Hologic, all the AC2 diagnostic Norwegian laboratories implemented supplementary testing using the Aptima CT (ACT) (Hologic) or GeneProof CT (GeneProof a.s., Brno, Czech Republic) assays, which both have different genetic targets and thus will detect the FI-nvCT. Additionally, as internationally recommended [4,5], AC2-negative/equivocal samples with RLUs between 15 and 99 and a positive result by supplementary testing (corresponding to ‘possible FI-nvCT cases’ [3,4]), were stored for subsequent CT 23S rRNA gene sequencing, which was performed as previously described [2,3].

**Figure 1:**
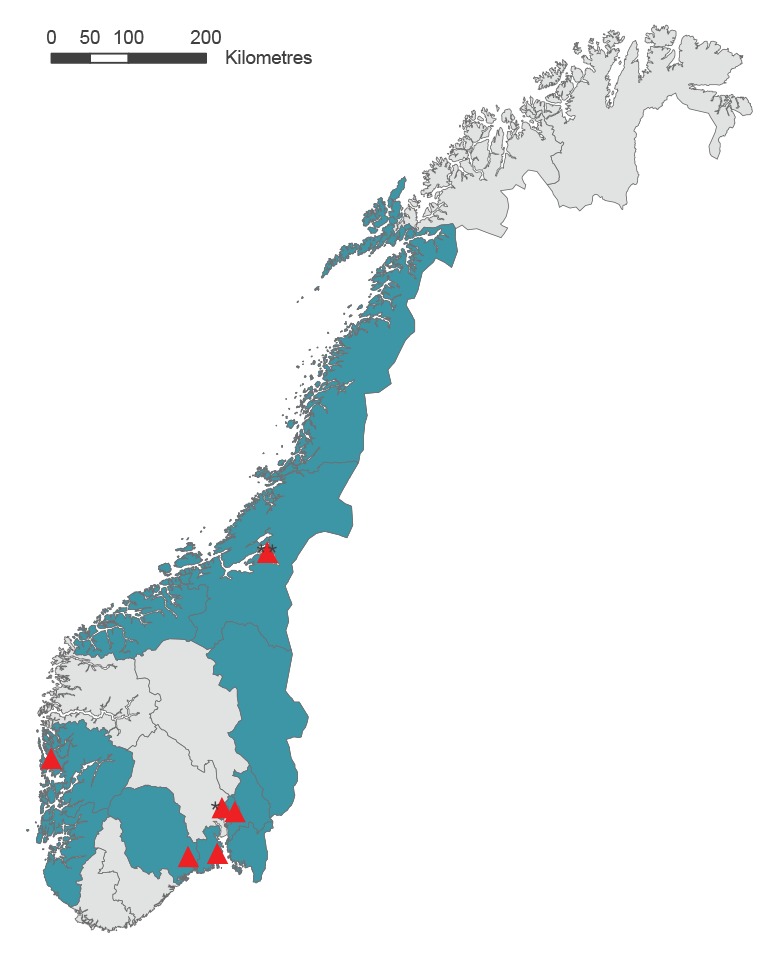
Counties of residence of patients diagnosed with the ‘Finnish new variant of *Chlamydia trachomatis*’ (FI-nvCT), Norway, June–August 2019 (n = 81 patients over 11 counties)

From June to August 2019, 97 samples from possible FI-nvCT cases [3,4] from five Norwegian laboratories were available for 23S rRNA gene sequencing. In 81 (84%) of these samples, the CT 23S rRNA C1515T mutation was detected, and accordingly those were confirmed as FI-nvCT cases [3,4]. The AC2 CT RLUs for the confirmed FI-nvCT samples ranged from 17 to 49, with 10% (8/81) of samples showing RLUs between 17 and 19 and 90% (73/81) RLUs between 20 and 49. These 81 confirmed FI-nvCT samples originated from 81 cases aged between 18 and 32 years (median age: 22 years), 67% (n = 54) of whom were women. The cases with the FI-nvCT originated from 11 of the 18 counties of Norway (Figure 1). Table 1 shows the number of samples positive for FI-nvCT per month of sampling, and the counties of residence for cases. No information regarding their sexual orientation or recent travel was available.

AC2: Aptima Combo 2.

Counties of residence of patients diagnosed with FI-nvCT are in blue colour. The location of the six laboratories that use the AC2 assay are marked in the map as red triangles. Asterisks indicate one laboratory, for which samples for 23S rRNA gene sequencing were unavailable (*) and one laboratory that started implementing AC2 testing in June 2019 (**).

**Table 1:** Monthly number of samples positive for ‘Finnish new variant *Chlamydia trachomatis*’ according to sequencing, with sex and county of residence of all the corresponding patients, Norway, June–August 2019 (n = 81 patients)

Month of sampling	Cases positive for FI-nvCT^a^	Female cases	Male cases	County of residence^b^
**June**	28	19	9	Ho, MR, O, V, Te, Ø
**July**	42	26	16	A, He, Ho, MR, N, O, R, Tr, V
**August**	11	9	2	A, O, R, Tr
**Total**	81	54	27	Not applicable

FI-nvCT: Finnish new variant *Chlamydia trachomatis*.

^a^ Not all Aptima Combo 2/Aptima *C. trachomatis* discordant samples were 23S rRNA gene sequenced during the period covered by the Table. Accordingly, these data cannot be used to describe any trends in the prevalence of FI-nvCT over time.

^b^ A: Akershus; He: Hedmark; Ho: Hordaland; MR: Møre and Romsdal; N: Nordland; O: Oslo; R: Rogaland; Te: Telemark; Tr: Trøndelag; V: Vestfold; Ø: Østfold.

## Recall of patients with previous Aptima Combo 2-negative/equivocal *Chlamydia trachomatis* results

Among the first 23S rRNA gene sequenced AC2/ACT discordant samples, we confirmed 28 of 37 samples as positive for FI-nvCT. These originated from cases residing in six different counties. Based on this high proportion and widespread geographic distribution of FI-nvCT, the Norwegian Institute of Public Health recommended a 6-month recall of patients for the laboratories using the AC2 test. The case definition for recall was a patient with a CT sample analysed after 1 January 2019 in one of the five Norwegian laboratories using the AC2 assay, where the sample had a negative or equivocal AC2 CT result and showing RLU values from 15 to 99. The sixth laboratory started using AC2 in June 2019, and recall was thus not relevant. In total, the five laboratories identified 1,360 samples in line with this case definition. One laboratory decided to extend the recall period back to July 2018 and, accordingly, 1,377 patients were offered re-testing. The results from re-testing of these recalled patients are pending.

## Retrospective analysis of Aptima Combo 2 *Chlamydia trachomatis* relative light units values and positivity rates

**Figure 2:**
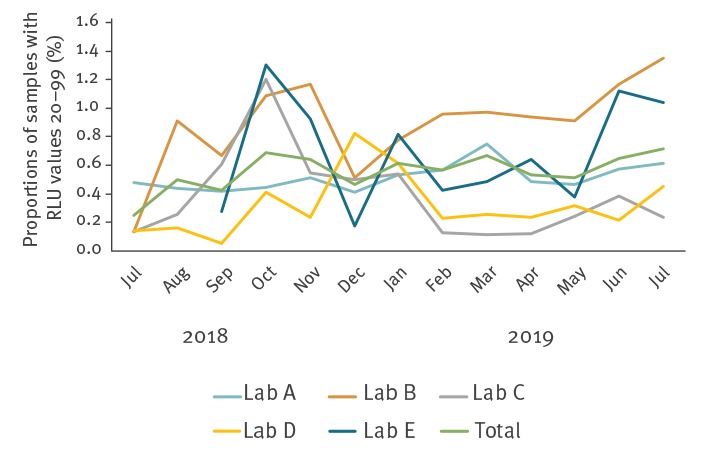
Proportions of samples yielding relative light unit values between 20 and 99 among all samples flagged as negative or equivocal for *Chlamydia trachomatis* by the Aptima Combo 2 assay in five diagnostic laboratories in Norway, July 2018−July 2019

To investigate the presence and spread of the FI-nvCT in Norway, AC2 CT test results were evaluated from the previous 13 months in accordance with recommendations by Unemo et al. [5]. Accordingly, information about the number and proportion of AC2 CT negative or equivocal specimens with elevated RLUs (15–99) among all specimens tested was collected from the five laboratories that routinely used AC2 in Norway from July 2018 to July 2019 as a proxy indicator for the possible presence of the FI-nvCT. The proportion of samples with RLU values between 15 and 19 among all AC2 CT negative samples varied substantially from month to month and between laboratories, and appeared in some cases to have been affected by time points for instrument service. On the contrary, the proportion of AC2 samples with RLUs between 20 and 99 among AC2 CT negative or equivocal samples appeared to be more robust for temporal trends. Figure 2 shows the proportions of specimens with AC2 CT RLUs between 20 and 99 from the five laboratories.

Lab: laboratory; RLU: relative light unit.

Laboratory A had the largest number of specimens (data not shown). The number of specimens analysed with negative or equivocal result in the other laboratories was as follows: 36,701 (Laboratory B), 11,250 (Laboratory C), 24,935 (Laboratory D), and 14,099 (Laboratory E, only data from September 2018 and onwards).

In brief, the proportions of AC2 CT negative or equivocal samples with RLU values 20–99 had slightly increased since July 2018 in all five laboratories, and a relatively large increase was observed in Laboratory B (from 0.1% to nearly 1.4%). However, the proportions substantially fluctuated over time and peaked at different time points, and the sample volume differed substantially in the laboratories (Figure 2). Accordingly, any observed trends must be interpreted with caution.

The AC2 CT positivity rates per month among AC2 processed samples in the five laboratories from July 2018 to July 2019 were also investigated, and showed that the overall CT positivity rates varied from 6.5% (1,072/16,505) to 7.3% (1,220/16,704). There was no obvious and consistent trend in the CT positivity rate over the analysed timespan for the laboratories overall. The positivity rates ranged from 4.8% (93/1,935) to 10.3% (154/1,497) for individual laboratories, and also for individual laboratories, the positivity rate varied from month to month and between laboratories without showing any clear and consistent trend. National CT surveillance data in Norway have shown that the CT positivity rate among all tested in Norway decreased from 9.1% (22,527/246,268) in 2010 to 6.8% (25,130/368,953) in 2017, but subsequently increased to 7.3% (26,570/362,603) in 2018 [7]. Apart from Laboratory C, all laboratories using AC2 also showed an increase in CT positivity rate from 2017 to 2018 (data not shown). The CT diagnosis rate per 100,000 inhabitants in Norway also increased from 2017 to 2018. The same was observed in Oslo and Hordaland, the two counties with the highest number of detected FI-nvCT. Surveillance data for 2019 are not yet available [7].

Most interestingly, two additional CT variants escaping detection by AC2 were also identified. One sample from June 2019 had a G1523A mutation in the CT 23S rRNA gene (AC2 RLU: 66) and one sample from July 2019 a C1514T mutation in the CT 23S rRNA gene (AC2 RLU: 15). These patients were between 20 and 29 years old and included one man and one woman. No information regarding sexual orientation or recent travel of these patients was available.

## Discussion

In the present paper, we provide evidence of the FI-nvCT in 11 of 18 Norwegian counties. This is the first published report of the FI-nvCT beyond Finland [1,2] and Sweden, where only two FI-nvCT specimens were reported in one Swedish county [3]. In Norway, the vast majority (84%) of the possible FI-nvCT samples, according to the case definition implemented by ECDC [4], exhibited the CT 23S rRNA C1515T mutation and accordingly were confirmed as FI-nvCT. Reflex testing with ACT (or other appropriate and quality-assured CT NAAT) of all AC2 samples showing CT RLUs 15–99 was implemented in June 2019, as advised by Hologic and internationally recommended [1-5]. Furthermore, a recall policy was timely implemented at all diagnostic laboratories using AC2 when the wide geographical spread within Norway was detected. This was performed to ensure that individuals who potentially had previously received false-negative AC2 CT results could be re-tested and, if CT positive, appropriately treated. The recommended recall period was 6 months, but one laboratory voluntarily increased this period to 12 months. This is also in concordance with our data indicating that the FI-nvCT may have been present in Norway before January 2019. A longer period for recall of patients nationally remains considered based on the retrospective local proportion of AC2 CT negative tests with RLUs 20–99, CT positivity rates, and the proportion of CT positives among the recalled and re-tested individuals (complete data pending). It is also important to consider other factors, such as the rate of spontaneous clearance of CT infection, re-testing already performed due to other reasons, potential risk of re-infection, and the social consequences for the patients [8-10]. At the current time point, we do not have data available to evaluate the impact of our strategy for recall of patients. This will be important to follow up in the near future.

The retrospective analysis of AC2 samples with CT RLUs between 20 and 99 and CT positivity rates indicated that it cannot be excluded that the FI-nvCT might have been present at least in some regions of Norway as early as July 2018 if not earlier. However, these data should be interpreted with great caution. Interestingly, the trends differed between the five laboratories, and the peak with the highest proportion of suspected FI-nvCT samples varied from October 2018 to July 2019. The five laboratories receive samples from different regions across Norway, and local variations in the spread of the FI-nvCT likely have affected the trends. Varying trends in positivity rates both for the FI-nvCT and CT in general could also be influenced by additional factors such as seasonal testing patterns and the case mix among the tested.

In the present paper, two additional CT variants (CT 23S rRNA C1514T and G1523A mutations) that resulted in false-negative CT tests in AC2 were detected. These mutations were found in single samples from July and June 2019, respectively. Both CT variants were also recently discovered in one sample each in June 2019 in the United Kingdom [11]. The Norwegian sample with the CT 23S rRNA C1514T mutation had a RLU value of only 15, which was lower than the RLU values detected for all the FI-nvCT samples (RLU: 17–49). The borderline RLU results suggest a possibility for diagnostic escape by mutants that may not be captured by the reflex testing algorithm for AC2 samples with CT RLU 15–99 [3-5]. It is of concern if such variants could be circulating internationally. More testing is required to assess if these two CT variants are circulating elsewhere in Norway, Europe, and globally. Due to the continuous evolution of CT, other false-negative CT variants can emerge (and likely already have) and subsequently spread [5]. This type of ‘diagnostically-selected’ evolution was also observed in 2006, when a new CT variant escaping detection in the Roche and Abbott NAATs available at that time was detected in Sweden. The variant subsequently spread to other countries, although this international spread remained limited [12,13].

In conclusion, the FI-nvCT, escaping detection in AC2, is widespread across Norway. The FI-nvCT has previously been detected in Finland [1,2] and Sweden [3]. This strongly emphasises the need for a European-wide, sufficiently powered and quality assured FI-nvCT study. The high number of FI-nvCT samples across Norway also indicates a long-term spread and it cannot be excluded that the FI-nvCT even emerged in Norway. Finally, the detection of the two additional rare CT variants (CT 23S rRNA C1514T and G1523A) that also escaped AC2 detection, illustrates the continuous evolution of CT creating new diagnostic-escape mutants. There is a requirement for an updated AC2 assay including a second genetic target sequence and a substantially improved surveillance for all types of CT variants and all types of CT diagnostic NAATs.
